# Immunological Studies on the Aerial Roots of the Indian Banyan

**DOI:** 10.4103/0250-474X.42970

**Published:** 2008

**Authors:** Tabassum Khan, Pratima Tatke, S. Y. Gabhe

**Affiliations:** C. U. Shah College of Pharmacy, S. N. D. T. Women's University, Juhu Tara Road, Santacruz (W), Mumbai-400 049, India

**Keywords:** *Ficus benghalensis*, hypersensitivity reaction, hemagglutination reaction, immunostimulant

## Abstract

The aqueous extract of the aerial roots the Indian Banyan, *Ficus benghalensis* L. (Family: Moraceae) was evaluated for its effect on both specific and non-specific immunity. This extract exhibited a significant increase in percentage phagocytosis by human neutrophils in the *in vitro* tests. It exhibited promising immunostimulant activity at doses of 50, 100, 200 and 400 mg/kg body weight in SRBC induced hypersensitivity reaction and hemagglutination reaction in rats. The aqueous extract was found to stimulate the cell mediated and antibody mediated immune responses.

Acquired immunodeficiency syndrome (AIDS) is one of the most threatening and rapidly spreading diseases in the world. It is caused by the human immunodeficiency virus (HIV). It infects and invades the cells of the immune system and renders the patient susceptible to opportunistic infections. The indigenous systems of medicine with tribal and folklore medicine have significantly contributed to the healthcare of the population of India especially in diseases where no satisfactory solutions are available in the allopathic system. Indian medicinal plants are a rich source of substances that are claimed to induce immunity^1^. Because the immune system is severely impaired once the HIV enters the body we decided to evaluate folk based medicines used as immune system boosters in the rural areas of Maharashtra, India.

An aqueous decoction of the fresh aerial roots of the Indian Banyan, *Ficus benghalensis* (Fam: Moraceae) has been used by Ayurvedic medical practitioners to boost the immune system in various diseases. However, no phytochemical and pharmacological investigations have been conducted so far. The current study is an effort to scientifically evaluate the immune boosting potential of the aqueous extract of the aerial roots of the Indian Banyan. This study also involved the determination of the phytoconstituents present in the aerial roots.

## MATERIALS AND METHODS

All the solvents used in the extraction process were of analytical grade. Minimum Essential Medium (MEM) used in the bioassay was procured from HiMedia Lab Pvt. Ltd. Ficoll Hypaque and bovine serum albumin were procured from Sigma Chemical Co. *Candida albicans* ATCC-10231 maintained on Sabourads agar (HiMedia) was used as the test microorganism in the bioassay. All the other reagents and chemicals used in the study were of analytical grade.

### Preparation of extracts:

The Indian banyan was authenticated at Agarkar's Research Institute, Pune, India. The growing tips of aerial roots were collected in the months of June to September from local areas of Mumbai. The root powder was extracted with distilled water using a Soxhlet extractor (hot solvent extraction method) for 18 h. The water was removed *in vacuo* (under reduced pressure) to give an aqueous extract of the roots. The aqueous extract was standardized with respect to the physico-chemical parameters such as consistency, pH and extractive value, water and alcohol soluble extractive values as per the Indian Pharmacopoeia^2^. Preliminary phytochemical screening of the aqueous extract was performed using qualitative chemical tests to identify the phytoconstituents present in the aqueous extract of the Indian Banyan^3^. The aqueous extract was evaluated for immunomodulatory activity using the *in vitro* polymorphonuclear (PMN) function test and *in vivo* animal experiments. The vehicle alone served as the control.

### Animals:

Random bred albino rats (male and female) reared in the C. U. Shah College of Pharmacy were used in the acute toxicity and pharmacological studies. The animals were maintained at room temperature and given a standard pellet diet (Lipton India Ltd) and tap water *ad libitum*. The test protocols for these studies were approved by the Institutional Animals Ethical Committee (CUSEP/IAEC/11/2003-2004 and CUSEP/IAEC/12/2003-2004)

### Antigen and polymorphonuclear leucocytes (PMN cells):

Sheep red blood cells (SRBCs) collected in Alsevier's solution washed in large volumes of sterile normal saline thrice and adjusted to a concentration of 5×10^9^ cells/ml with saline were used for immunization and challenge. Polymorphonuclear leucocytes (PMN cells) collected from normal healthy volunteers (age group 18-22 y) with no evidence of bacterial, fungal or viral infection were used in the study.

### Separation of peripheral blood mononuclear cells (PBMCs):

PBMCs were separated from the blood sample of K3 EDTA containing bulb by using the Ficoll Hypaque density gradient separation. 5 ml of whole blood was overlayed on 5 ml of Ficoll Hypaque-1077 gradient (Sigma). This was then centrifuged at 1500 rpm for 15 min. The buffy coat layer of PBMCS was isolated and washed once with sterile RPMI-1640 medium. The cells were then cryopreserved at a density of 2×10^6^ cells/ml using RPMI-1640 medium, 10% fetal calf serum and 10% DMSO till further use.

### *In vitro* phagocytosis test:

The aqueous extract was evaluated for immunomodulatory activity using the PMN function test. Peripheral venous blood, 10 ml, was collected from volunteers in a sterile heparinised tube. Neutrophils were isolated by Ficoll Hypaque density gradient sedimentation^4^. The RBC-PMN pellet was then subjected to dextran sedimentation. The supernatant containing more than 90% of PMN cells was collected and the cell density was adjusted to 1×10^6^ cells/ml using MEM.

*Candida albicans* (cell density adjusted to 1×10^6^ cells/ml using MEM) was used as the test microorganism. The PMN cells (cell density adjusted to 1×10^6^ cells/ml using MEM) were mixed with 1×10^6^ cells/ml of *Candida albicans* and incubated at 37º for 1 h in 5% CO_2_ atmosphere, in presence of the test extracts. The control was identical solution minus the test extracts. Cytosmears were prepared after incubation. The smear was fixed with methanol, stained with Giemsa stain and observed under 100 × oil immersion objectives to determine the phagocytic activity of the PMN cells. Neutrophils (100 nos.) were scanned and the cells with ingested microorganisms were counted^5^. The parameters evaluated were percentage phagocytosis (percentage of PMN cells involved in phagocytosis) and phagocytic index (ratio of number of *Candida albicans* engulfed to the total number of cells involved in phagocytosis). The percentage immunostimulation was calculated by using the formula^6^, % Immunostimulation= (Phagocytic index_TEST_ –Phagocytic index_CONTROL_/Phagocytic index_CONTROL_) × 100.

### Acute toxicity and hypersensitivity reactions:

The acute toxicity study for the aqueous extract was conducted as per the prescribed guidelines for the testing of chemicals^7^. Hypersensitivity reaction to SRBC was induced in rats following the method of Doherty^8^. The aqueous extract (in doses of 50, 100, 200 and 400 mg/kg) was administered to the animals (the test group) orally for five days and vehicle was administered to the control animals. The aqueous extract was administered orally on each of the two days prior to immunization, on the day of immunization and on each of the two days after immunization. (i.e. days -2, -1, 0, +1. +2).

The rats were immunized by injecting 0.1 ml of SRBC solution subcutaneously into the right hind footpad on day 0. The animals were challenged seven days later by injecting the same amount of SRBC into the left hind footpad. Thickness of the left hind footpad was measured with a micrometer at 4 h and 24 h after challenge.

### Hemagglutination reaction:

The aqueous extract (in doses of 50, 100 mg, 200 and 400 mg/kg) was administered to the animals (the test group) orally for five days and vehicle was administered to the control animals. The extract was administered orally on each of the two days prior to immunization, on the day of immunization and on each of the two days after immunization. (i.e. days -2, -1, 0, +1. +2).

**Fig. 1:**
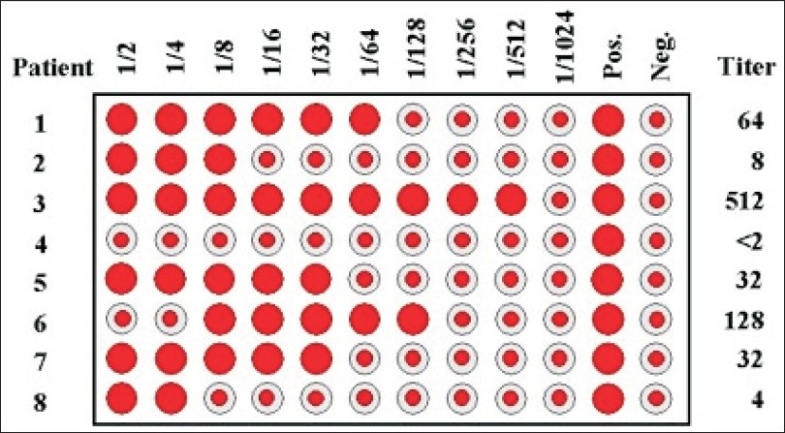
Demonstration of hemagglutination using antibodies against sheep red blood cells The 96 well microplate shows the serum dilution and calculation of antibody titer in the hemagglutination assay.

The rats were immunized by injecting 0.5 ml of SRBC solution intraperitoneally on the day of immunization. Blood samples were collected from the animals by retro-orbital puncture on the tenth day after immunization. Antibody levels were determined by the hemagglutination technique^9^. Twofold serial dilution was performed of one volume (100 μl) of serum sample with one volume of 0.1% bovine serum albumin (100 μl). One volume (100 μl) of 0.1% SRBCs in BSA in saline was added and the tubes were mixed thoroughly. They were allowed to settle down at room temperature for about 60 to 90 minutes until the control tube showed unequivocally negative pattern (a small button formation). The value of the highest serum dilution showing visible hemagglutination was taken as the antibody titer (fig. 1).

### Lymphocyte proliferation assay:

Peripheral blood lymphocytes (PBL) were obtained from the buffy coat residues by the Ficoll Hypaque method. Under sterile conditions, 50 μl of PBL suspension (5×10^6^ cells/ml), 50 μl sample dilutions and 50 μl of Phytohemagglutinin (PHA) (5 μg/ml) were mixed in sterile 96 well flat-bottomed microtitre plates. The plates were incubated at 37º in a 5% CO_2_ incubator for 48-72 h. After incubation cell growth was quantitated using MTT. For this 25 μl of MTT (1 mg/ml) was added to each well after which the plates were incubated at 37° for 4 h. Next 50 μl of acid propanol (0.04 M HCl in isopropanol) was added and the contents of each well mixed thoroughly. Plates were read on an automated ELISA reader at 540 nm. Controls consisted of PBL with PHA (100% activity), PBL with RPMI (0% activity) and sample with PHA (background)^10^. Test wells contained 50 μl of PBMC's+50 μl of PHA+50 μl of sample, background wells contained 50 μl of PHA+50 μl of sample and the control wells used to calculate 100% activity: -50 μl of PBMC's + 50 μl of PHA and 0% activity: - 50 μl of PBMC's + 50 μl of RPMI

### Statistical analysis:

The data was analyzed using one way analysis of variance (ANOVA) followed by Dunnett's ‘t’ test with the level of significance set at P < 0.05.

## RESULTS AND DISCUSSION

The aqueous extract was standardized with respect to physico-chemical parameters viz. color, consistency, pH and extractive value (Table 1). Qualitative chemical tests were carried out to determine the presence of phytoconstituents. The aqueous extract was found to contain flavonoids, phenolics, saponins, proteins and carbohydrates. The results of the acute toxicity study indicated that the LD_50_ of the aqueous extract of *Ficus benghalensis* was 2500 mg/kg body weight.

**TABLE 1:** PHYSICO-CHEMICAL EVALUATION OF THE AQUEOUS EXTRACT

Physicochemical properties	Aqueous extract
Nature	
	Semisolid
Colour	
	Dark brown
pH	
	10.0
Extractive value %w/w	
	24.37%
Alcohol soluble extractive of the root powder %w/w	8%
Water soluble extractive of the root powder %w/w	25%

The results are an average of three estimates

Immunomodulatory agents are the substances that modify the host immune response. Polymorphonuclear (PMN) leucocytes and circulating monocytes form the first line of host defense by virtue of their ability to phagocytose invading microorganisms.

The aqueous extract exhibited a dose dependent stimulation of phagocytosis of *Candida albicans* by the human neutrophils (Table 2). It resulted in a significant increase in percentage phagocytosis at concentrations of 0.5 mg/ml (55%), 1.0 mg/ml (64%) and 2.0 mg/ml (54%) versus 34% in the control. The maximum stimulation of phagocytosis by human neutrophils was observed at a concentration of 1 mg/ml. It exhibited a 25% immunostimulation at this concentration. Increasing the concentration to 2 mg/ml resulted in a decrease in percentage phagocytosis. This is in agreement with the dose-activity relationships of immunomodulatory substances. As a rule it can be stated that good immunostimulants are active in only low concentrations, at high concentrations they can cause a decrease or reversal i.e. immunosuppressive effects^11^.

**TABLE 2:** *IN VITRO* PHAGOCYTOSIS TEST OF THE AQUEOUS EXTRACT

Test extract	Concentration (mg/ml)	% Phagocytosis	Phagocytic index	% Immunostimulation
Aqueous extract	Control	34±1.11	1.60±0.04	
	0.5	55±2.80*	1.93±0.06	21.05±6.22
	1.0	64±1.45*	2.01±0.03*	25.35±2.03
	2.0	54±1.78*	1.86±0.05*	16.04±4.38

The results are an average of six estimates. The values are expressed as mean±SEM; Significance levels

* P<0.05 (Dunnets‘t’ test) versus the vehicle control.

Hypersensitivity reaction is an exaggerated immune response that results in tissue damage and is manifested on subsequent contact with an antigen. Such reactions are classified as early (4 h) and delayed (24 h) type of hypersensitivity reactions depending on the time taken for the onset of action. Since the aqueous extract exhibited immunostimulant activity at 1 mg/ml in the *in vitro* test, we thought it prudent to increase the oral dose by 50-100 fold in the *in vivo* studies. Hence we conducted the *in vivo* experiments at 50, 100, 200 and 400 mg/kg.

Per oral administration of the aqueous extract for five days produced a dose related increase in early (4 h) and delayed (24 h) hypersensitivity reactions in rats (Table 3). The maximum response was observed at a dose of 100 mg/kg. To confirm the effect of the aqueous extract on the cellular immune response, we evaluated the proliferation of human lymphocytes in response to PHA. The results indicated an increase in proliferation with increasing concentrations of the test extract with the maximum response (83.57% proliferation) at 1 μg/ml.

**TABLE 3:** HYPERSENSITIVITY REACTION OF THE AQUEOUS EXTRACT

Aqueous extract (Oral dose in mg/kg)	Inflammation after challenge Difference in rat paw thickness before and after giving the antigen in mm	
	
	4 h	24 h
Control	0.45±0.02	0.03±0.13
50	0.88±0.12*	0.51±0.17*
100	1.48±0.19*	0.76±0.16*
200	1.03±0.16*	0.79±0.15*
400	1.28±0.15*	0.14±0.18

The results are an average of six animals. The values are expressed as mean±SEM; Significance levels

* P<0.05, (Dunnets‘t’ test) versus the vehicle control.

Humoral antibody response is mediated by antibody produced by B-lymphocytes. The antigen antibody reaction results in agglutination. The relative strength of an antibody titer is defined as the reciprocal of the highest dilution that is still capable of causing visible agglutination. The antibody titer is useful to measure the changes in the amount of the antibody in the course of an immune response. Per oral administration of the aqueous extract for five days produced a dose related increase in the antibody titer in rats (Table 4). The maximum response was observed at a dose of 100 mg/kg.

**TABLE 4:** HEMAGGLUTINATION REACTION OF THE AQUEOUS EXTRACT

Aqueous extract (Oral dose in mg/kg)	Hemagglutination antibody titer	
	
	Range	Mean Antibody titer
Control	128 - 256	205±14.02
50	512 - 2048	1126±112.17*
100	2048 - 4096	2458±183.17*
200	1024 - 2048	1638±112.17*
400	1024 - 2048	1638±112.17*

The results are an average of six animals. The values are expressed as mean±SEM Significance levels

* P<0.05, (Dunnets‘t’ test) versus the vehicle control.

It is believed that the human health is related to immune responses^12^. The immune cells and their mediators are directly involved in the processing of antigens, removal of microorganisms by phagocytosis, lysis of bacteria and viruses. There are no previous reports on the immunostimulant properties of the aerial roots of *Ficus benghalensis.* In the present study, the aqueous extract of the aerial roots of *Ficus benghalensis* was found to stimulate the non-specific arm of immunity as evidenced by the increase in the phagocytic function and efficiency of the neutrophils treated with the test extract versus the control. The aqueous extract was found to enhance both cells mediated and antibody-mediated immune responses as well as phagocytosis. The aqueous extract was found to stimulate the lymphocyte proliferation by 86% versus the control thereby confirming the hypothesis that it enhances the cell-mediated immunity. The maximum response in both the *in vivo* studies was observed at 100 mg/kg. Increasing the dose beyond 100 mg/kg resulted in a decrease in the immune stimulation response.

The activation and proliferation of T-lymphocytes and cytokine production post stimulation with antigens play important roles against bacterial and viral infection. Immune stimulation is important in many disease conditions where there is a suppression of normal immune responses viz. AIDS. In AIDS, the HIV infects the T-helper cells that are the central controllers of immune responses to antigens. When these cells become infected with HIV, many other cell types fail to function normally and this results in a severe depression of the immune system. The body becomes highly susceptible to infections with opportunistic pathogens eventually leading to death. Hence immune restoration becomes imperative in the overall management of AIDS because death eventually is a result of failed immune system rather than the virus itself. Thus improving immune health is an important part of a long-term strategy for managing AIDS.

The aqueous extract of *Ficus benghalensis* was found to stimulate both cell and antibody mediated immune responses. It also stimulates proliferation lymphocytes that are responsible for orchestrating an immune response to an antigen/disease. Hence the aqueous extract of *Ficus benghalensis* has a strong potential to be explored further as an immune-based therapy along with antiHIV drugs in the overall management of AIDS.
